# Supplementary material for: Interactive Effects of Copper Pipe, Stagnation, Corrosion Control, and Disinfectant Residual Influenced Reduction of Legionella pneumophila during Simulations of the Flint Water Crisis
Source: Pathogens. 2020 Sep 4;9(9):730. doi: 10.3390/pathogens9090730 (PMC7559348; doi:10.3390/pathogens9090730)
Supplement: Supplementary file 1 [file pathogens-09-00730-s001.pdf]

**Table S1.** PCR confirmation of unique morphologies that were identified morphologically as *Legionella*. Positives, indicated by an "+", are colonies counted as *Legionella* and correctly identified by PCR amplification of identification gene markers. Negatives, indicated by an "0", are plates that contained no colonies morphologically identified as *Legionella* or were not confirmed by PCR. Lspp = *Legionella spp*, LP= *Legionella pneumophila*, SG1 = *Legionella pneumophila* serogroup 1. When available, all visually unique colonies were picked for colony PCR confirmation from plates during each sampling event (n = 20-12 colonies per sampling).

| **Conditions** | **Inoculation** | | | **Day 47** | | | **Day 82** | | | **Day 175** | | | **Day 210** | | |
|---|---|---|---|---|---|---|---|---|---|---|---|---|---|---|---|
| | ***L* spp** | ***LP*** | ***SG1*** | ***L* spp** | ***LP*** | ***SG1*** | ***L* spp** | ***LP*** | ***SG1*** | ***L* spp** | ***LP*** | ***SG1*** | ***L* spp** | ***LP*** | ***SG1*** |
| **FR** | + | + | + | + | + | 0 | + | + | 0 | + | + | + | + | + | + |
| *FR-CC* | + | + | + | + | + | 0 | + | + | 0 | + | + | 0 | + | + | 0 |
| *FR-NoFe* | + | + | + | + | + | 0 | + | + | 0 | + | + | + | + | + | 0 |
| *DET* | + | + | + | 0 | 0 | 0 | + | + | 0 | + | + | + | + | + | 0 |
| **DET-Cold** | + | + | + | + | + | 0 | + | + | 0 | + | + | 0 | + | + | 0 |
| **DET-Enhanced** | + | + | + | 0 | 0 | 0 | + | + | 0 | 0 | 0 | 0 | 0 | 0 | 0 |
